# Perianal fistulizing Crohn's disease is associated with a higher prevalence of HPV in the anorectal fistula tract. A comparative study

**DOI:** 10.1016/j.clinsp.2023.100219

**Published:** 2023-05-31

**Authors:** Lucas Rodrigues Boarini, Carlos Walter Sobrado, Giana Rabello Mota, Luisa Lina Villa, Idblan Carvalho de Albuquerque, Natalia Sousa Freitas Queiroz, Carolina Bortolozzo Graciolli Facanali, Sidney Roberto Nadal, Ivan Cecconello

**Affiliations:** a Departamento de Gastroenterologia, Hospital das Clínicas, Faculdade de Medicina da Universidade de São Paulo, São Paulo, SP, Brazil; b Centro de Investigação Translacional em Oncologia, Faculdade de Medicina da Universidade de São Paulo, São Paulo, SP, Brazil; c Departamento de Coloproctologia, Heliópolis Hospital, São Paulo, SP, Brazil; d Instituto de Infectologia Emilio Ribas, São Paulo, SP, Brazil

**Keywords:** Crohn's Disease, HPV (Human Papilloma Virus), Anal Cancer

## Abstract

**Background & Aims:**

Perianal fistulizing Crohn's disease is the main risk factor for anal cancer in patients with inflammatory bowel disease. Whether this occurs due to a higher frequency of human papillomavirus remains unclear. The authors aimed to evaluate the prevalence of HPV and high-risk HPV in patients with perianal Crohn's disease, compared with a control group.

**Methods:**

The authors conducted a two-center cross-sectional study in which perianal fistulizing Crohn's disease patients were matched for age and sex with patients with anorectal fistula without Crohn's disease. Biopsy specimens were obtained from fistulous tracts during examination under anesthesia for both groups. The samples were sent for HPV detection and genotyping using the INNO-LiPA test.

**Results:**

A total of 108 subjects (54 in each group) were recruited. The perianal fistulizing Crohn's disease group showed a statistically higher frequency of HPV in the fistulous tract than the control group (33.3% vs. 16.7%; p = 0.046). Separate analyses on high-risk types demonstrated that there was a numerically higher frequency of HPV in the perianal fistulizing Crohn's disease group. In multiple logistic regression, patients with perianal fistulizing Crohn's disease were found to have a chance of HPV 3.29 times higher than patients without Crohn's disease (OR = 3.29; 95% CI 1.20‒9.01), regardless of other variables. The types most frequently identified in the perianal fistulizing Crohn's disease group were HPV 11 (12.96%) and HPV 16 (9.26%).

**Conclusion:**

Perianal fistulizing Crohn's disease is associated with a higher prevalence of HPV than in patients with anorectal fistula without Crohn's disease.

## Highlights

• Perianal Crohn's disease is the main risk factor for anal cancer in IBD patients.
• Perianal Crohn's disease patient has a chance of HPV 3.29 time higher than control.
• High risk HPV is numerically twice as prevalent in perianal Crohn's disease patient.

## Introduction

Perianal Fistulizing Crohn's Disease (PFCD) is a disabling complication present in 23% to 38% of patients with Crohn's Disease (CD) and is considered to be an independent factor for poor prognosis [1,2]. Optimal management of perianal fistulas remains challenging and often requires multidisciplinary assessment. The gold-standard treatment, consisting of a combination of anti-TNF medication and passage of a seton has only been capable of inducing complete perianal remission in 52.6% of the patients [3]. This shows that almost half of the patients with PFCD will continue to have disease activity, with the risk that, over time, this inflammation may cause anatomical changes that compromise evacuative physiology and lead to perineal sepsis, chronic pain, and anal cancer [4,5]. The yearly incidence of anal Squamous Cell Carcinoma (SCC) in PFCD is 0.26/1000 patient-years (95% CI 0.03‒0.92), which is approximately 20 times higher than in the general population [6,7].

HPV is a small non-enveloped DNA virus with icosahedral symmetry [8,9]. Certain factors hinder the clearance of the virus and promote its persistence in the body, thereby increasing the risk of lesions of the lower genital tract, including intraepithelial neoplasms and carcinomas. Early sexual initiation, anal intercourse, multiplicity of sexual partners, smoking, associated Sexually Transmitted Infections (STI), and primary and acquired immunodeficiencies have been reported as risk factors for the occurrence of lower genital tract lesions 10, 11, 12. Ruel et al. evaluated patients with Inflammatory Bowel Disease (IBD) who developed neoplastic squamous lesions of the anal canal (SCC, high and low-grade intraepithelial neoplasia, and Small-Cell Carcinoma). Approximately 83% of the lesions observed were related to Human Papillomavirus (HPV) infection, mainly by oncogenic subtypes 16 and 18. All patients who developed anal canal SCC had PFCD. Therefore, in inflammatory bowel diseases, the appearance of squamous neoplastic changes seems to be related to HPV, and the development of SCC seems to be related to the occurrence of PFCD [13].

PFCD is the main risk factor for the development of anal squamous cell carcinoma in inflammatory bowel diseases [13,14]. It is not yet known whether this positive association results from chronic inflammation of the perianal region or from a higher prevalence of HPV since the frequency of HPV in this population is not well established. The aim of this study was to compare the prevalence of HPV in a group of patients with perianal fistulizing Crohn's disease and a control group.

## Materials and method

This was a two-center cross-sectional study that was developed at Hospital das Clínicas, University of São Paulo Medical School, and Heliópolis Hospital. These two institutions are referral centers for treating PFCD in the city of São Paulo, Brazil. The authors follow the STROBE Statement to guide the present study's manuscript.

The patients in the PFCD group were recruited between December 2018 and November 2020 at the inflammatory bowel disease outpatient clinic, after being electively indicated for seton or fistulotomy procedures. The control group consisted of patients with perianal fistula without CD who were selected at the general coloproctology outpatient clinic. The following patients were excluded from the study: under-eighteens; pregnant women; Human Immunodeficiency Virus (HIV)-positive individuals; patients presenting immunosuppression due to other pathological conditions or medications; patients with transplants; patients with previous histories of radiotherapy or surgical treatment for SCC; patients with a previous or current history of anogenital HPV; patients undergoing HPV vaccination schemes; patients who did not agree to participate in the study; and those who did not sign the free and informed consent statement. This project was approved by the Ethics Committee for the Analysis of Research Projects (CAPPesq) and Ethics Committee of Heliopolis Hospital on 11/07/2018, with a Certificate of Presentation for Ethical Assessment 00265418.0.0000.0068.

Data collection took place in three steps and was performed by a single physician. The first step consisted of an interview, to obtain the patients’ clinical characteristics. The second step consisted of a review of the medical records to confirm the diagnosis and staging of Crohn's disease. The third step consisted of filling out a printed questionnaire by the patient addressing sexual behavior.

### Sample size

To determine the sample size, the prevalence of anal HPV in the general population was considered to be around 5% [15]. There are no data in the literature on the prevalence of HPV among patients with PFCD. Thus, the authors estimated that in the population with perianal Crohn's disease, the proportion would be about five times higher, taking HIV infection as the reference point, given that this is the immunosuppressive condition that has been most studied in relation to the prevalence of HPV.

The sample size obtained was 110 patients, with 55 individuals in each group, considering a loss/refusal potential of greater than 10%, with a statistical significance level of 5% and test power of 80%.

### Material analyzed and blinding

**Fig. 1:**
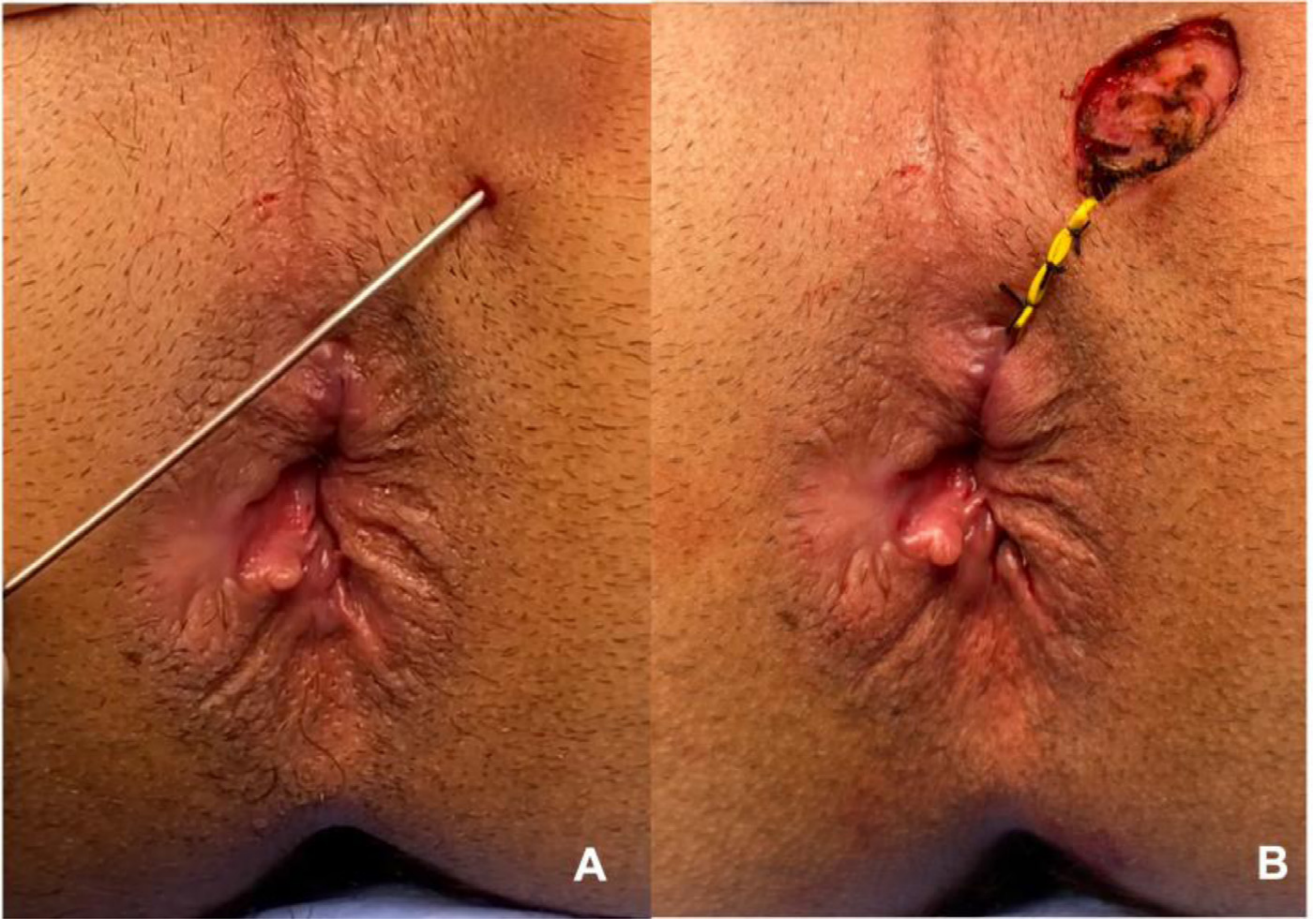
This figure illustrates how the extraction of the analyzed sample was performed. (A) Demonstrates the identification of the external fistulous orifice and (B) Demonstrates the resected external orifice that was sent for HPV research.

The material analyzed was obtained during the surgical procedure for treating perianal fistula (fistulotomy or seton placement). The external orifice and/or path of the fistula was biopsied and sent for analysis (Fig. 1). The material was embedded in paraffin blocks, and these were sectioned by a single biomedical technician, following a careful protocol to avoid contamination. This technician was unaware of the group to which the material belonged, thus respecting the rules for blinding between groups. Four sections per block (per patient) were obtained. The first of these was mounted on a slide and stained with hematoxylin-eosin to identify the presence of squamous stratified tissue, which was always done by the same pathologist, for all samples. The next three sections were placed in Eppendorf tubes for subsequent genotyping and identification of the virus. To process each successive paraffin block, the technician changed gloves, replaced the cutting knife, and cleaned the entire surface that the block would come into contact with, using 70% alcohol, to avoid contamination of the sample. The histological material was selected and evaluated by the Pathology Department of Heliópolis Hospital. Following paraffin removal and proteinase K digestion, DNA was purified from the tissue sections by phenol-chloroform extraction and ethanol precipitation.

Detection of the virus and genotyping was performed using the INNO-LiPA™ HPV genotyping extra kit (Fujirebio©), which can identify 32 types of HPV by means of a PCR reaction followed by reverse hybridization, as described below. Part of the L1 region of the HPV genome was amplified by means of PCR using biotinylated SPF10 (65 bp) primers, and using the human HLA-DPB1 gene, as quality control for the samples. This assay allows simultaneous and independent detection of 13 high-risk HPV genotypes (16, 18, 31, 33, 35, 39, 45, 51, 52, 56, 58, 59 and 68), six of probable high-risk (26, 53, 66, 70, 73 and 82), nine of low risk (6, 11, 40, 42, 43, 44, 54, 61 and 81) and four that are not classified regarding risk (62, 67, 83 and 89). All the manufacturer's instructions were followed for testing. For HPV genotyping, the automated equipment AutoLipa48® (Fujirebio*®*, Belgium) was used.

### Statistical analysis

The qualitative characteristics were evaluated using absolute and relative frequencies, and associations of the characteristics in relation to the groups were verified, using the chi-square test or exact tests (Fisher's exact test or likelihood ratio test). The quantitative characteristics were described according to groups, using summary measurements (mean, standard deviation, median, minimum, and maximum), and were compared between groups using the Student *t* test or the Mann-Whitney test.

The presence of HPV was described in accordance with the characteristics that have been shown to be related to HPV in the literature. Associations of qualitative characteristics with the presence of HPV were verified using chi-square tests or exact tests. The quantitative characteristics were compared according to the presence of HPV by using the Student *t* test or the Mann-Whitney test.

Unadjusted Odds Ratios (OR) were estimated for each characteristic evaluated for the chances of occurrence of HPV, using bivariate logistic regression and estimated multiple logistic regression models. The authors selected the variables that in the bivariate tests presented significance levels lower than 0.20 (*p* < 0.20) and with clinical relevance for explaining the presence of HPV in patients, and all variables were maintained in the final model (full model).

The IBM-SPSS for Windows software, version 20.0, was used to perform the analyses and the Microsoft Excel 2003 software was used to tabulate the data; p-values below 0.05 were considered statistically significant.

## Results

A total of 110 patients were recruited over a two-year period (2018 to 2020): 55 patients in the PFCD group and 55 patients in the control group. One patient in the PFCD group was excluded because it was found that he had previously undergone treatment for a condylomatous lesion, and one patient in the control group was excluded because he received a confirmed HIV diagnosis just a few months after sample collection and would thus have been in the immunological window. Hence, 108 patients remained in the study for the final analysis, comprising 54 patients in each group.

Among the demographic and behavioral characteristics evaluated, only schooling presented a statistically significant difference between the groups (p = 0.024), such that the patients with PFCD had higher levels of education than the patients in the control group (Table 1). Variables such as age, gender, color, marital status, smoking, previous anal sex, number of sexual partners, previous STIs, men who have sex with men and comorbidities were similar in the two groups evaluated.

**Table 1:** Personal characteristics, behaviors, and results of analyzes not adjusted between groups.

Variable	Total	Group		*p*
	(*n* = 108)	Control (*n* = 54)	PFCD (*n* = 54)	
**Age (years)**				0.986^a^
Mean, y (range)	34.5 (18; 67)	34 (18; 66)	35.5 (18; 67)	
**Gender, *n* (%)**				>0.999
Male	52 (48.1)	26 (48.1)	26 (48.1)	
Female	56 (51.9)	28 (51.9)	28 (51.9)	
**Race, *n* (%)**				0.294^b^
White	52 (48.1)	22 (40.7)	30 (55.6)	
Mixed	45 (41.7)	26 (48.1)	19 (35.2)	
Black	10 (9.3)	5 (9.3)	5 (9.3)	
Asian	1 (0.9)	1 (1.9)	0 (0)	
**Scholarity, *n* (%)**				**0.024**
Elementary School	35 (32.4)	23 (42.6)	12 (22.2)	
Complete high school or higher	73 (67.6)	31 (57.4)	42 (77.8)	
**Marital status, *n* (%)**				0.335
Maried/Cohabited	51 (47.2)	28 (51.9)	23 (42.6)	
Single/Separated/Divorced	57 (52.8)	26 (48.1)	31 (57.4)	
**Smoking status, *n* (%)**				0.326^b^
No	82 (75.9)	38 (70.4)	44 (81.5)	
Current smoker	26 (24.1)	16 (29.6)	10 (18.5)	
**Anal sex, *n* (%)**	26 (24.1)	15 (27.8)	11 (20.4)	0.368
**Men who have sex with men, *n* (%)**	8 (15.4)	4 (15.4)	4 (15.4)	>0.999^c^
**Sexual partners, *n* (%)**				0.504
0 to 2	29 (26.9)	11 (20.4)	18 (33.3)	
3 to 4	29 (26.9)	16 (29.6)	13 (24.1)	
5 to 10	31 (28.7)	17 (31.5)	14 (25.9)	
> 10	19 (17.6)	10 (18.5)	9 (16.7)	
**Comorbities, *n* (%)**	31 (28.7)	12 (22.2)	19 (35.2)	0.136
**Sexually transmitted infectious disease ever, *n* (%)**	12 (11.2)	8 (14.8)	4 (7.5)	0.234

Qui-Square test.

a Student *t* test.

b Likelihood ratio test.

c Fischer's exact test.

Regarding the specific clinical characteristics of the PFCD group, the mean age was 35.5 years, 51.9% were female, and the time since receiving the diagnosis of the disease was a mean (SD) of 7.76 (6.58) years. A total of 90.7% of the patients were undergoing anti-TNF treatment at the time of sample collection; 7.4% were being treated with ustekinumab; 1.9% were being treated with vedolizumab; and 29.62% had previously been exposed to at least one biological therapy. Concomitant use of azathioprine was identified in 48.2% of the patients and only two patients were using corticosteroids. The vast majority (88.8%) of the patients presented colonic disease in association with perianal disease, 96.3% of the patients had a history of perianal surgery, with an average of 4.09 ± 3.6 operations per patient (Table 2).

**Table 2:** Specific characteristics of patients with PFCD.

Variable	Total
	(*n* = 54)
**Duration of perianal disease (years)**	
Mean (SD)	7.76 ± 6.58
**Current medication, *n* (%)**	
Adalimumab	19 (35.2)
Infliximab	19 (35.2)
Certolizumab	11 (20.4)
Ustekinumab	4 (7.4)
Vedolizumab	1 (1.9)
Combotherapy with Azathioprine	26 (48.2)
Corticosteroid dependence	2 (3.7)
One biologic failure	9 (16.7)
Two biologic failure	3 (5.6)
Three biologic failure	4 (7.4)
**Montreal Age, *n* (%)**	
A1	8 (14.8)
A2	36 (66.7)
A3	10 (18.5)
**Montreal Behavior, *n* (%)**	
B1	30 (55.5)
B2	17 (31.5)
B3	7 (13)
**Montreal Location, *n* (%)**	
L1	5 (9.3)
L2	26 (48.1)
L3	22 (40.7)
L4	1 (1.9)
**Previous perianal surgery, mean (SD)**	4.09 ± 3.6
**History of seton procedure, *n* (%)**	43 (79.6)
**History of abdominal surgery, *n* (%)**	14 (25.9)
Total colectomy	1 (1.9)
Enterectomy or stricture plasty	3 (5.6)
Segmentar colectomy	3 (5.6)
Ileocolectomy	8 (14.8)
**Ostomy, *n* (%)**	
Current ostomy	4 (7.4)
Ostomy closure	4 (7.4)

Table 3 shows that the patients with PFCD presented a higher frequency of HPV in the material collected from the fistulous tract (33.3% vs. 16.7%; p = 0.046), in relation to the control group. The separate analyses on high-risk HPV subtypes showed that was numerically twice as frequent in the PFCD group, but this was not statistically significant (18.5% vs. 9.3%; p = 0.164).

**Table 3:** Comparation of HPV frequency between groups and results of unadjusted analyses.

Variable	Total	Group		*p*
	(*n* = 108)	Control (*n* = 54)	PFCD (*n* = 54)	
**HPV**				**0.046**
No	81 (75)	45 (83.3)	36 (66.7)	
Yes	27 (25)	9 (16.7)	18 (33.3)	
**Hign risk**				0.164
No	93 (86.1)	49 (90.7)	44 (81.5)	
Yes	15 (13.9)	5 (9.3)	10 (18.5)	
**Potential high risk**				>0.999^a^
No	101 (93.5)	51 (94.4)	50 (92.6)	
Yes	7 (6.5)	3 (5.6)	4 (7.4)	
**Low risk**				0.139
No	95 (88)	50 (92.6)	45 (83.3)	
Yes	13 (12)	4 (7.4)	9 (16.7)	

Qui-Square test

a Fischer's exact test.

Through analyzing the frequency of HPV and adding the variable PFCD into the analysis as a potential risk factor together with factors known to be influential such as the number of previous partners, previous STI, anal sex, age, gender, marital status, and smoking, it could be seen that only the presence of PFCD and the number of partners influenced the presence of HPV statistically (p = 0.046 and p = 0.006 respectively) (Table 4).

**Table 4:** Results of unadjusted analyzes to demonstrate the relationship of characteristics of interest with the presence of HPV.

Variable	HPV		OR	CI (95%)		*p*
	No (*n* = 81)	Yes (*n* = 27)		Inferior	Superior	
**Group, *n* (%)**						**0.046**
Control	45 (83.3)	9 (16.7)	1			
PFCD	36 (66.7)	18 (33.3)	2.5	1	6.23	
**Age (years)**			0.98	0.94	1.02	0.365^a^
Average (mín.; máx.)	35 (18; 67)	34 (19; 54)				
**Gender, *n* (%)**						0.657
Male	40 (76.9)	12 (23.1)	1			
Female	41 (73.2)	15 (26.8)	1.22	0.51	2.93	
**Marital status, *n* (%)**						0.738
Maried/Cohabited	39 (76.5)	12 (23.5)	1			
Single/Separated/Divorced	42 (73.7)	15 (26.3)	1.16	0.48	2.79	
**Scholarity, *n* (%)**						0.553
Elementary School	25 (71.4)	10 (28.6)	1			
Complete high school or higher	56 (76.7)	17 (23.3)	0.76	0.31	1.89	
**Smoking status, *n* (%)**						0.436
No	60 (73.2)	22 (26.8)	1			
Yes	21 (80.8)	5 (19.2)	0.65	0.22	1.93	
**Sexually transmitted infectious disease ever, *n* (%)**						0.493^b^
No	72 (75.8)	23 (24.2)	1			
Yes	8 (66.7)	4 (33.3)	1.57	0.43	5.68	
**Anal sex, *n* (%)**						0.194
No	64 (78)	18 (22)	1			
Yes	17 (65.4)	9 (34.6)	1.88	0.72	4.93	
**Sexual partners, *n* (%)**						**0.006**^c^
0 to 2	25 (86.2)	4 (13.8)	1			
3 to 4	26 (89.7)	3 (10.3)	0.72	0.15	3.55	
5 to 10	20 (64.5)	11 (35.5)	3.44	0.95	12.45	
> 10	10 (52.6)	9 (47.4)	5.63	1.41	22.53	

Qui-Square test.

a Student *t* test.

b Fischer`s exact test.

c Likelihood ratio test.

When the authors translocated the factors with positive associations with HPV in a multiple logistic regression table (Table 5), the authors observed that patients with PFCD had a 3.29 times higher chance of HPV than patients without Crohn's disease (OR = 3.29; 95% CI 1.20‒9.01). Together, patients with more partners over the course of their lives had a higher chance of HPV, and the chance of HPV in patients with five to ten partners was 4.25 times higher than the chance for patients with up to two partners, while in patients with more than ten partners, the chance of HPV was 6.86 times higher.

**Table 5:** Result of the multiple models to explain HPV prevalence.

Variable	OR	IC (95%)		*p*
		Inferior	Superior	
Group (PFCD)	3.29	1.2	9.01	**0.02**
Anal Sex	1.24	0.41	3.74	0.702
Sexual partners (0 to 2)	1			
3 to 4	0.85	0.17	4.32	0.846
5 to 10	4.25	1.09	16.66	**0.038**
> 10	6.86	1.52	31.02	**0.012**

Multiple logistic regression.

**Fig. 2:**
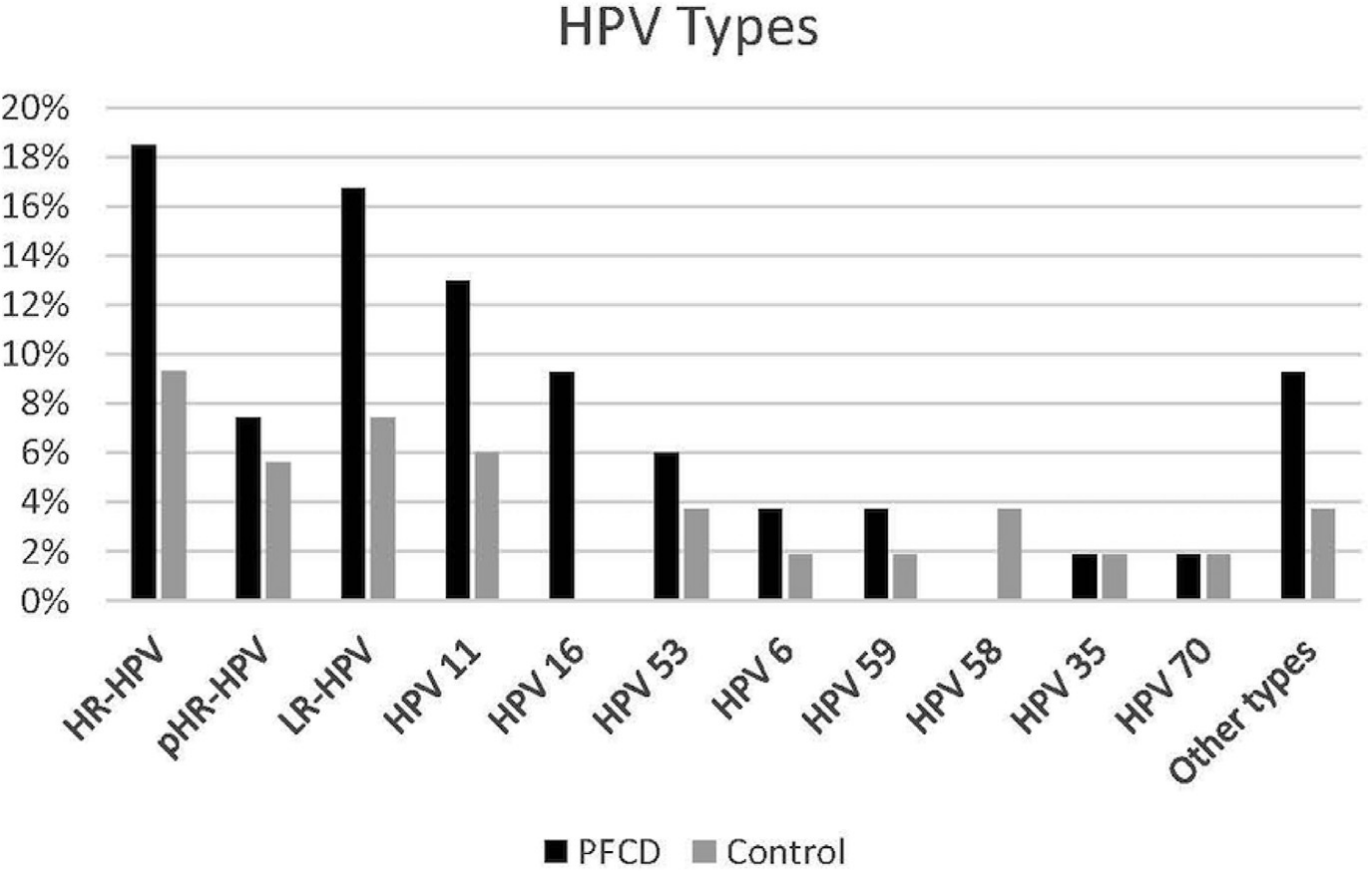
Perianal fistulizing Crohns disease group presented at least one type of high-risk HPV in 18.5%, among which HPV 16 was the most frequent type (9.26%), 7.40% had potential high-risk HPV and 16.70% had low-risk HPV, among which HPV 11 was the most prevalent, identified in 12.96% of the patients with PFCD.

Specific analysis on the HPV types showed that in the PFCD group, 18.5% presented at least one type of high-risk HPV, among which HPV 16 was the most frequent type (9.26%), 7.40% had potential high-risk HPV and 16.70% had low-risk HPV, among which HPV 11 was the most prevalent, identified in 12.96% of the patients with PFCD (Fig. 2).

## Discussion

The present study demonstrated that PFCD is an independent factor for the higher prevalence of HPV in relation to a control group: One-third of the patients with PFCD (33.3%) presented HPV in the perianal fistulous tract and 18.5% of them had high-risk HPV. The frequency of high-risk HPV was approximately twice that found in the control group, but this difference was not statistically significant. A larger sample would be needed to assess whether the higher frequency of high-risk HPV becomes significant at the population level. Some studies have suggested that PFCD is the main risk factor for anal cancer in patients with IBD [6,13,14]. However, it remains unknown whether this positive association results from a higher prevalence of HPV or from a local inflammatory state that was exacerbated by the presence of perianal fistulas. Given that the frequency of HPV in this population is unknown, the present study brings insightful information to fill in this gap in the literature.

HPV 16 and 18 are the main subtypes correlated with anal carcinogenesis and are responsible for 87% of anal cancer cases worldwide [16]. Li et al. have shown that the frequency of subclinical cervical infection by HPV 16 and 18 in Chinese women was higher in patients with IBD than in controls (7.3% vs. 0.3%; *p* < 0.001) [17]. Accordingly, the PAPILLAN study evaluated 469 patients who underwent colonoscopy under anesthesia and collected anal canal material for HPV investigation through the INNO-LiPA kit. It was found that 101 (21.5%) of these patients presented IBD. It was demonstrated that patients with CD were more frequently affected by high-risk HPV than the general population (30.0% vs. 18.1%; p = 0.005; and 14.0% vs. 6.8%; p = 0.007), and subtype 16 was the most frequently observed. In a sub-analysis of 22 patients with perianal Crohn's disease, eleven had HPV in the anal canal (50%) vs. 24/79 (30.4%) among other patients with IBD (p = 0.12). However, the small sample size of Crohn's disease patients in this study prevents further extrapolations of the findings for all patients with PFCD [18].

It is speculated that in chronic and systemic inflammatory states, the presence of complex perianal fistulas requiring multiple local procedures along with the use of immunomodulatory and/or immunosuppressive drugs may affect the defense mechanisms responsible for viral clearance, thereby promoting maintenance of HPV in fistulous pathways. Permanent high-risk HPV infection can initiate a carcinogenic cascade by preventing apoptosis of dysplastic cells, with consequent malignancy. The authors tried to minimize the differences between the groups by comparing the patients with PFCD with patients with anorectal fistula unrelated to CD. Thus, although perianal inflammation is generally higher in CD, both groups had anorectal fistulas.

The potential role of biological therapy and immunosuppressive treatments on the prevalence of HPV in the IBD population is unknown. It has been demonstrated that chronic viral infections, such as varicella-zoster virus and hepatitis B virus can be reactivated with immunosuppression [19]. Notably, a recent population-based French study showed that thiopurine monotherapy demonstrated an increased risk of opportunistic viral infection compared with antitumor necrosis factor monotherapy [20]. Conversely, studies comparing the use of biological and immunosuppressive medications in relation to nonuse of these medications have not observed any higher prevalence of HPV, high-risk HPV, or altered anal cytology in patients using these medications, in relation to controls [18,21,22]. It is important to emphasize that all the patients in the PFCD group were being treated with biological therapy in association or not with azathioprine, which could have influenced the prevalence of HPV in this population.

Diagnostic screening methods for identifying HPV and cytological alterations have been used since the 1960s. Anal brush cytology is the method most used, given its low cost and high feasibility [23]. Despite its benefits, it presents some limitations in the anal region: its sensitivity and specificity for detecting dysplasia have varied greatly between different studies, ranging from 47% to 90%, and 16% to 92%, respectively [24]. In addition, it only presents moderate agreement (66%), in interobserver analysis using the Bethesda criteria [25]. For these reasons, the authors chose to perform the HPV test through the INNO-LiPA kit, because this method provides greater sensitivity in analyses on formalin-fixed, paraffin-included tissues, and also because it allows evaluation of fistulous tissue. However, it has the disadvantage of higher costs in relation to cytology.

Historically, patients with inflammatory bowel disease have been found to have higher schooling levels and greater purchasing power than the general population. The authors found higher schooling in the PFCD group than in the control group, in line with the literature [26].

Prophylactic vaccines have been shown to be effective in the primary prevention of HPV infection and its associated diseases [27,28]. However, to date, there is no mandatory recommendation for patients with PFCD, because the true prevalence of the virus in this population is not well established in the literature. Wisniewski et al published a large review study on anal neoplasia in IBD cases. They advocated vaccination in this population, as recommended by the European Crohn's and Colitis Organization, along with the development of screening measures for anal cancer in PFCD patients [14,29]. As such, the findings of the present study strongly support the recommendation of HPV vaccination for all PFCD patients.

The present study is associated with some limitations. Firstly, the cross-sectional design is methodologically unequipped to address any risk correlation between the higher frequency of HPV and the occurrence of anal cancer. Moreover, information regarding sexual behavior may be subject to information bias due to embarrassment. The authors tried to overcome this bias by handing the questionnaire while the patients were in the waiting room, in order to avoid oral questioning. Finally, the authors believe the small sample size of this study is a major limitation to further extrapolations of the authors’ findings.

## Conclusion

In our study, it can be concluded that the patients with perianal fistulizing Crohn's disease had a higher prevalence of HPV than patients with perianal fistulas without Crohn's disease. Further prospective studies are warranted to assess the long-term impact of HPV infection on the risk of anal cancer in PFCD patients.

## Authors’ contributions

Boarini L.R. coordinated the study, he contributed to the conceptualization, project administration, and writing original draft. Sobrado CW was involved in the study conceptualization and supervision. Mota G.R. and Villa L.L. contributed in methodology and investigation. Albuquerque I.C. biopsied the fistulous tract during the surgical procedure and contributed to investigation. Queiroz N.S.F. contributed to writing review and editing the manuscript. Facanali C.B.G., Nadal S.R., and Cecconello were involved in the conception and writing review. All authors contributed to the critical revising and the final approval of the manuscript.

## Declaration of Competing Interest

The authors declare no conflicts of interest.
